# Supplementary material for: Pdx1 Is Transcriptionally Regulated by EGR-1 during Nitric Oxide-Induced Endoderm Differentiation of Mouse Embryonic Stem Cells
Source: Int J Mol Sci. 2022 Apr 1;23(7):3920. doi: 10.3390/ijms23073920 (PMC8999925; doi:10.3390/ijms23073920)
Supplement: Supplementary file 1 [file ijms-23-03920-s001.zip › Suplementary Figures.pdf]

Supplementary Figures

Figure S1

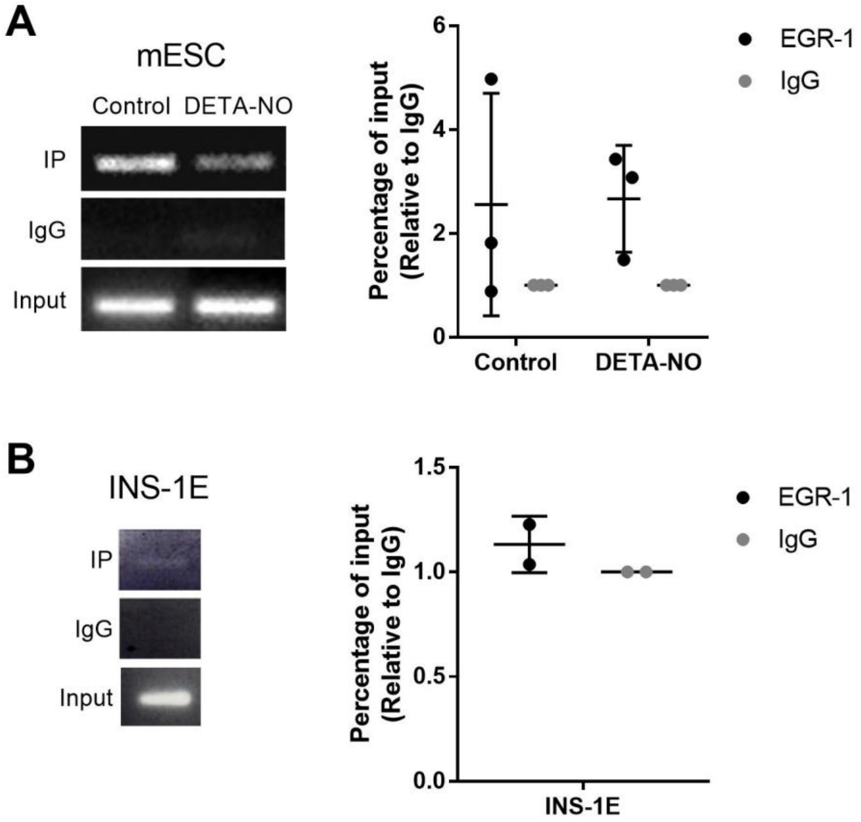

Supplementary Figure 1. Chromatin immunoprecipitation (ChIP) assays of EGR-1 on *Pdx1* promoter at regulatory area III in (**a**) mESCs and (**b**) INS-1E cell line. The figure shows (left panels) two representative ChIP results by qualitative PCR and (righ panels) the means ± SD of two (a) and three (b) independent experiments. The Y axis corresponds to the percent input relativized to IgG binding.

Figure S2

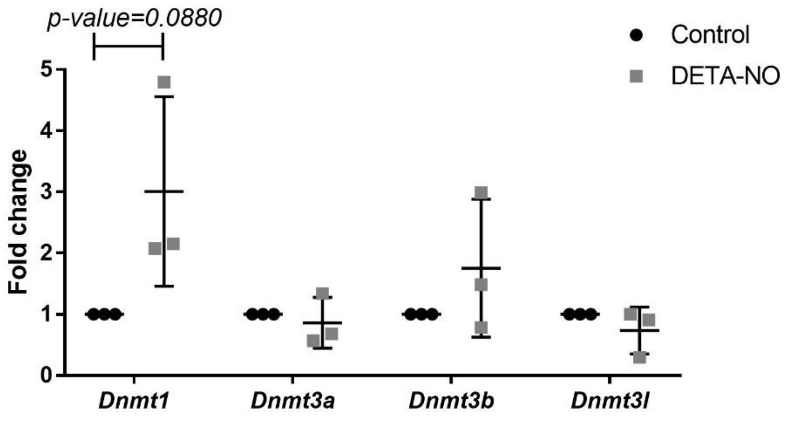

Supplementary Figure 2. DNA methyltransferase expression in mESCs. Analysis of DNA methyltransferases (Dnmt) expression after DETA-NO treatment by real time-PCR. These values were normalized to the expression values of the *β-Actin*, used as loading control. The data is analyzed using ΔΔCt algorithm and relativized to control condition. They represent the average of three independent experiments. Data are means ± Standard deviation (SD).

Figure S3

A) Control condition

Methylation

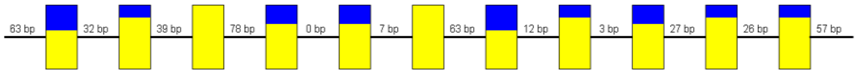

CpG Position	1	2	3	4	5	6	7	8	9	10	11
Unmethylated	4.0	2.0	0.0	3.0	3.0	0.0	4.0	2.0	3.0	2.0	2.0
Methylated	6.0	8.0	10.0	7.0	7.0	10.0	6.0	8.0	7.0	8.0	8.0
Not present	0	0	0	0	0	0	0	0	0	0	0

Legend: ■ unmethylated ■ methylated ■ not present

B) DETA-NO condition

Methylation

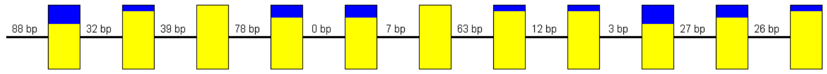

CpG Position	1	2	3	4	5	6	7	8	9	10	11
Unmethylated	3.0	1.0	0.0	2.0	2.0	0.0	1.0	1.0	3.0	2.0	1.0
Methylated	7.0	9.0	10.0	8.0	8.0	10.0	9.0	9.0	7.0	8.0	9.0
Not present	0	0	0	0	0	0	0	0	0	0	0

Methylation

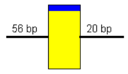

CpG Position	12
Unmethylated	1.0
Methylated	9.0
Not present	0

Legend: ■ unmethylated ■ methylated ■ not present

C) Technical control: Untreated DNA and analysis of *Pdx1* proximal CpG Island

Methylation

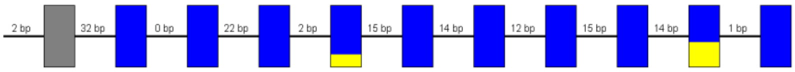

CpG Position	1	2	3	4	5	6	7	8	9	10	11
Unmethylated	0.0	5.0	5.0	5.0	4.0	5.0	5.0	5.0	5.0	3.0	5.0
Methylated	0.0	0.0	0.0	0.0	1.0	0.0	0.0	0.0	0.0	2.0	0.0
Not present	5	0	0	0	0	0	0	0	0	0	0

Methylation

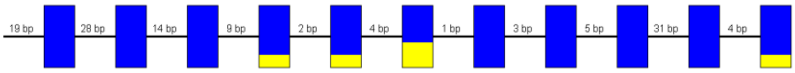

CpG Position	12	13	14	15	16	17	18	19	20	21	22
Unmethylated	5.0	5.0	5.0	4.0	4.0	3.0	5.0	5.0	5.0	5.0	4.0
Methylated	0.0	0.0	0.0	1.0	1.0	2.0	0.0	0.0	0.0	0.0	1.0
Not present	0	0	0	0	0	0	0	0	0	0	0

Methylation

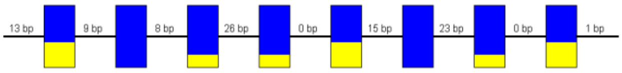

CpG Position	23	24	25	26	27	28	29	30
Unmethylated	3.0	5.0	4.0	4.0	3.0	5.0	4.0	3.0
Methylated	2.0	0.0	1.0	1.0	2.0	0.0	1.0	2.0
Not present	0	0	0	0	0	0	0	0

Legend: ■ unmethylated ■ methylated ■ not present

D) Technical control: DNA treated with the CpG Methyltransferase, M.SssI and analysis of Pdx1 proximal CpG Island

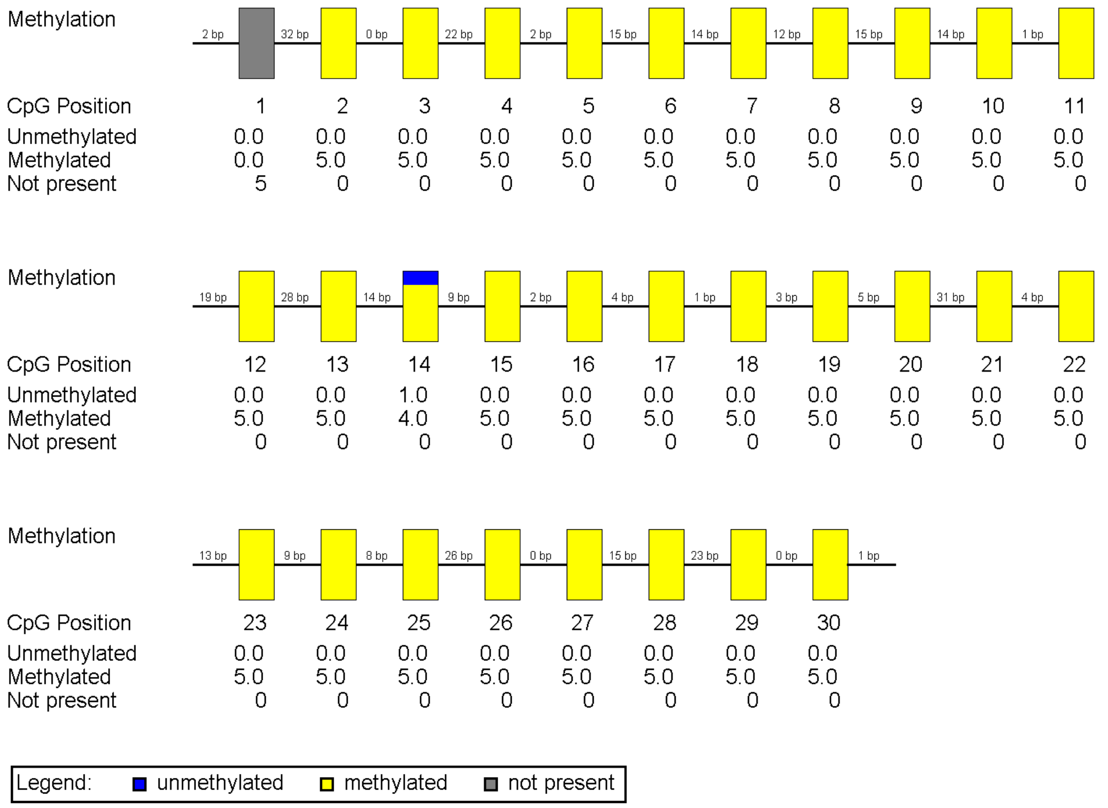

Supplementary Figure 3. Methylation analysis of *Pdx1* promoter by bisulfite sequencing PCR (BSP). The graphs show the CpG sites studied, which are represented by vertical rectangles. The methylation status is represented by the color of the rectangles: yellow-methylated and blue-unmethylated. The number of methylated or unmethylated CpG sites found in each condition is detailed under each rectangle. Graphs show the methylation results of four Pdx1 promoter regions or cell culture conditions: EGR-1 binding site in mESCs cultured in control (A) and (B) DETA-NO conditions; and *Pdx1* proximal CpG Island in mEScs cultured in control condition, untreated (C) and treated with CpG Methyltransferase, M.SssI (D).
